# Omni-dimensional dynamic convolution feature coordinate attention network for pneumonia classification

**DOI:** 10.1186/s42492-024-00168-5

**Published:** 2024-07-08

**Authors:** Yufei Li, Yufei Xin, Xinni Li, Yinrui Zhang, Cheng Liu, Zhengwen Cao, Shaoyi Du, Lin Wang

**Affiliations:** 1 School of Information Science and Technology, Northwest University, Xi’an, 710127 Shaanxi Province China; 2 Department of Ultrasound, the Second Affiliated Hospital of Xi’an Jiaotong University, Xi’an, Shaanxi Province 710004 China; 3 National Key Laboratory of Human-Machine Hybrid Augmented Intelligence, National Engineering Research Center for Visual Information and Applications, and Institute of Artificial Intelligence and Robotics, Xi’an Jiaotong University, Xi’an, Shaanxi Province 710049 China

**Keywords:** Pneumonia, Coordinate attention, Dynamic convolution, ResNet18, X-ODFCANet

## Abstract

Pneumonia is a serious disease that can be fatal, particularly among children and the elderly. The accuracy of pneumonia diagnosis can be improved by combining artificial-intelligence technology with X-ray imaging. This study proposes X-ODFCANet, which addresses the issues of low accuracy and excessive parameters in existing deep-learning-based pneumonia-classification methods. This network incorporates a feature coordination attention module and an omni-dimensional dynamic convolution (ODConv) module, leveraging the residual module for feature extraction from X-ray images. The feature coordination attention module utilizes two one-dimensional feature encoding processes to aggregate feature information from different spatial directions. Additionally, the ODConv module extracts and fuses feature information in four dimensions: the spatial dimension of the convolution kernel, input and output channel quantities, and convolution kernel quantity. The experimental results demonstrate that the proposed method can effectively improve the accuracy of pneumonia classification, which is 3.77% higher than that of ResNet18. The model parameters are 4.45M, which was reduced by approximately 2.5 times. The code is available at https://github.com/limuni/X-ODFCANET.

## Introduction

Pneumonia is an acute respiratory infection caused by viruses, bacteria, or fungi that result in inflammation of the lungs and interstitial lung changes, leading to lung-tissue damage and even death. Pneumonia is the leading cause of death among children under the age of five, kills more children than any other infectious disease, claiming the lives of over 700,000 children under 5 years old annually, or approximately 2,000 children every day [1, 2]. Among individuals over the age of 75 years, pneumonia is also a significant contributor to mortality, with an estimated 100,000 deaths annually [3]. COVID-19 is a novel coronavirus that began to break out in 2019 and spread worldwide, becoming a danger to human health. The virus is transmitted between people through respiratory droplets or close contact with contaminated surfaces [4]. Infected people develop symptoms such as fever, cough, and respiratory distress 2-14 days after exposure to the virus [5]. Therefore, timely detection and treatment are of great importance in patients with pneumonia.

Currently, chest X-ray (CXR) [6], computed tomography (CT) [7], and magnetic resonance imaging (MRI) [8] are commonly used in hospitals for the diagnosis of pneumonia. Doctors generally make a comprehensive diagnosis of COVID-19 by combining antigen testing, nucleic-acid testing, CXR, CT, and MRI. The Zhongnan Hospital of Wuhan University suggested that pneumonia may be diagnosed by detecting clinical symptoms as well as radiological manifestations of pneumonia [9]. In addition, Ai et al. [10] showed that CT has high sensitivity for the diagnosis of COVID-19 and can be used as the primary diagnostic tool. Despite the high sensitivity of CT in the detection of chest-film abnormalities [11], the use of this method still faces some challenges; for example, CT scanners are non-portable and require sterilization of the device and imaging room after patient use. Moreover, the radiation dose is considerably higher than that of X-rays [12], and CT is not recommended for children as they are more sensitive to radiation. Compared with CT, portable CXR equipment is readily available, inexpensive, and available in most primary-care hospitals [13]. Additionally, CXR can be performed in isolated rooms; therefore, their use in hospitals reduces staff exposure to viruses and the risk of infection [14]. Therefore, CXR is considered the main diagnostic method for the clinical diagnosis and management of children with suspected COVID-19 [15, 16].

The current diagnostic approach to pneumonia mainly relies on the subjective experience of clinicians, and the clinical symptoms of COVID-19 are very similar to those of pneumonia caused by viruses or bacteria. Some patients present only mild symptoms; thus, distinguishing COVID-19 from other common types of pneumonia caused by the respiratory tract is difficult using clinical features alone [17]. Accordingly, computer-aided diagnosis has played an important role in medical research, clinical disease diagnosis, and treatment in recent years, for example, in the early detection of arthritis and chest diseases [18, 19].

Convolutional neural networks are particularly adept at classification tasks, but their performance in pneumonia classification is constrained by the fixed nature of the convolution kernels. This hinders their ability to adapt to complex feature extraction, and the lack of a clear channel-importance weighting presents a challenge. To overcome these issues in the classification of pneumonia, this study proposes X-ODFCANet, which has the ability to comprehend spatial information and enables flexible adaptive convolution kernels to classify pneumonia by inputting CXRs. First, ResNet18 is used as the backbone to effectively avoid gradient-explosion and gradient-disappearance problems caused by an increase in the number of middle layers of the network [20]. Second, the feature coordinate attention (FCA) module added to the network enhances the localization of significant features in the image and highlights the significance of spatial information. This enables the model to better comprehend and utilize the spatial structure of an image without introducing excessive parameters. Additionally, it enables the network to handle images effectively with multiscale information and improves the generalization ability of the model. Third, the omni-dimensional dynamic convolution (ODConv) module embedded in our network allows the model to adjust the convolution kernel in different dimensions to capture rich information and better capture long-range dependencies in the input data. Its dynamic nature enables the network to select and adjust the convolution kernel automatically during the learning process, thereby ensuring computational efficiency. Finally, the effectiveness of the method is verified using a public dataset. Experiments show that our proposed method can classify pneumonia with high accuracy and a low number of parameters.

The main contributions of this study are as follows:

- A network named X-ODFCANet is proposed for pneumonia classification. The integration of the FCA module enhances feature localization and spatial information, improves spatial-structure comprehension without introducing excessive parameters, and enhances the network generalization.
- The integration of the ODConv module facilitates adaptive convolution-kernel adjustments in different dimensions, capturing rich information and long-range dependencies, while maintaining computational efficiency.
- Experiments on the public dataset show that our proposed method can classify pneumonia with high accuracy and a low number of parameters.

### Attention mechanism

**Fig. 1:**
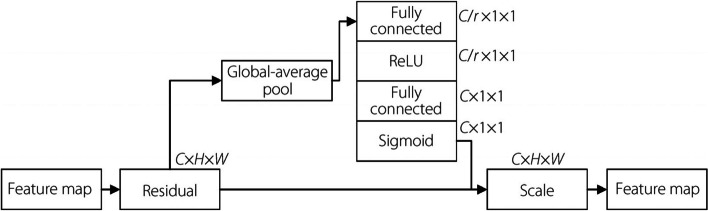
SE module structure

ResNet treats all feature channels equally without differentiating the importance of each channel. This approach can dilute the focus on channels that may carry more relevant information for specific tasks, such as pneumonia detection, where certain features, e.g., textures and shapes of lung opacities, are more informative than others. Attention mechanisms are generally integrated into either the channel or spatial dimensions. Hu et al. [21] proposed a channel attention module, squeeze-and-excitation (SE) module. In Fig. 1, the operation of the SE module is divided into the following two parts. (1) First is the squeeze operation, which compresses the spatial dimension of the input features through a global pooling layer. The channel dimensions are then flattened and reduced by a fully connected (FC) layer. (2) An excitation operation, nonlinear function, and activation function are used to increase the dimensionality to ensure the consistency of the input and output channels and obtain the channel attention feature map. Finally, convolution is performed with the original output, that is, the reweighting of the SE module is completed.

**Fig. 2:**
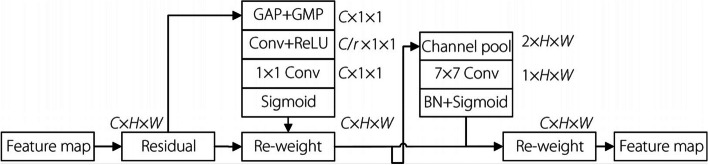
CBAM module structure

However, because the SE module only considers the adjustment of the channel dimension and not the spatial dimension, the extracted feature information loses its spatial part. This problem is solved by the proposed convolution block attention module (CBAM) [22], which focuses on information not only on the channel but also on the spatial dimension. It performs the attention-weighting operation from both the channel and spatial dimensions to obtain feature information containing both spatial and channel dimensions. The network structure of the CBAM module is schematically illustrated in Fig. 2.

### Dynamic convolution

**Fig. 3:**
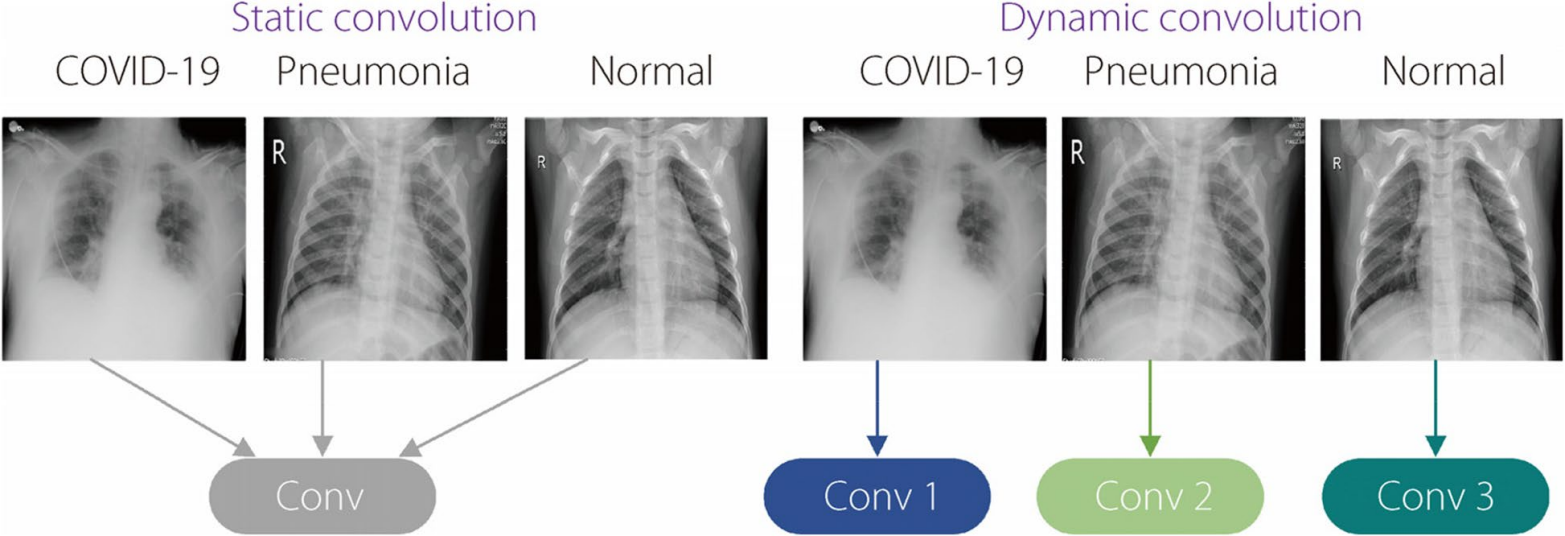
Static convolution *vs* dynamic convolution

Conventional convolution has the feature of weight sharing; that is, all samples share convolution parameters in the convolution network. Therefore, to increase the capacity of the model, the depth or width of the network must be increased, which leads to an increase in the computation complexity and number of parameters of the model, making model deployment difficult. However, in some practical-application scenarios, a high real-time performance of the model is required, as well as the need for a model with a low number of parameters and computations. To solve these problems, Chen et al. [23] proposed a dynamic convolution mechanism that could increase the expressiveness of a model without increasing the depth or width of the network. Dynamic convolution works by continuously and automatically adjusting the convolution parameters according to the input image. It makes adjustments for different images (e.g., viral pneumonia, COVID-19, and normal) and processes them using more suitable convolution parameters. A comparison of static and dynamic convolution is presented in Fig. 3. In the static convolution, the convolution kernel does not depend on the input function, whereas in the dynamic convolution, the convolution kernel is the input function.

**Fig. 4.**
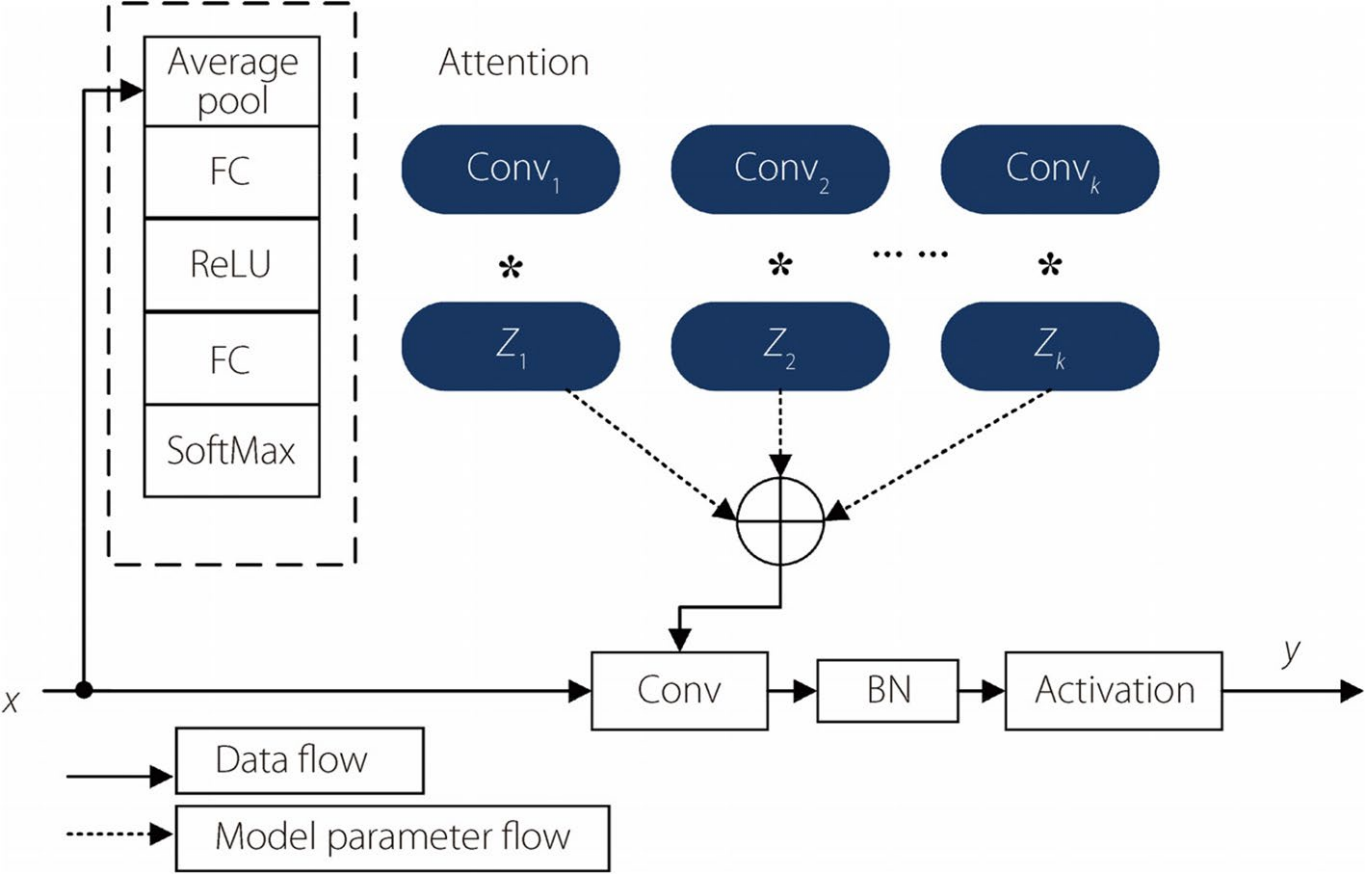
Dynamic convolution structure

Unlike static convolution, which uses a single kernel, dynamic convolution can be combined into a dynamic kernel based on the generation of multiple dynamically parallel convolution kernels that are extremely data-dependent and can dynamically adjust the weights of the dynamic convolution kernels depending on the input data to improve the generation and expression of the network. The dynamic-convolution process is shown in Fig. 4.

As shown in Fig. 4, in the dynamic-convolution structure diagram, the input data *x* is first passed through the attention module containing the average pooling, the FC layer, the rectified linear unit (ReLU) layer, the FC layer, and the Softmax function to obtain the dynamic convolution kernel weight parameters with data dependencies. Second, it is multiplied by the corresponding initialized convolution-kernel parameters separately to obtain a dynamic convolution kernel containing data dependencies using a weighting operation. Finally, the normalization and activation-function operations are performed. In summary, the entire process of dynamic convolution introduces only a small amount of extra computation through the attention mechanism and weighting operation of the convolution kernel to improve network performance.

## Methods

### Overall network design

**Fig. 5:**
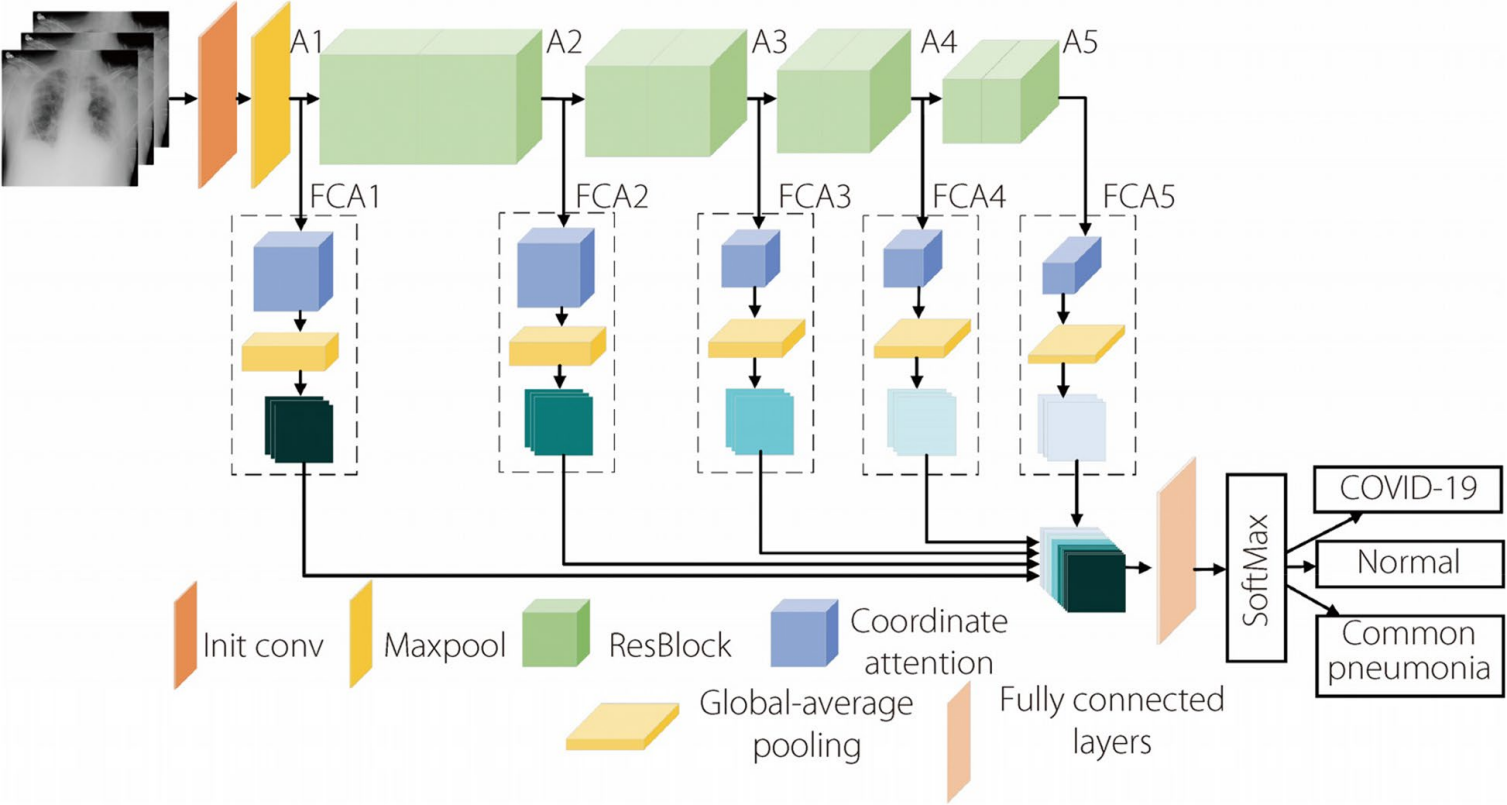
X-ODFCANet structure

In the process of feature extraction for chest radiographs, detailed features such as textures are easily ignored because of the insufficient directional feature extraction by deep learning. Therefore, an X-ray omni-dimensional dynamic convolution feature-coordinate attention (CA) network named X-ODFCANet is proposed. By adding the FCA and ODConv modules to the residual network, the ability of the network to extract feature information from chest radiographs is enhanced. A diagram of the specific network structure is presented in Fig. 5.

The model comprises the following five components: an initial convolution layer, a maximum pooling layer, an ODConv residual module, a feature-coordinated attention module, and a FC layer. The specific configuration of inception modules is shown in Table 1.

**Table 1:** Layer structure of X-ODFCANet

Layer	Output size	ODCAResNet
Convolution	$112\times 112$	$7\times 7\text{, }64\text{, stride 2}$
Maxpooling	$56\times 56$	$3\times 3\text{, stride 2}$
Layer1	$56\times 56$	$$\left[\begin{array}{c}1\times \text{1, 5}\\ 1\times \text{1, 4}\end{array}\right]\times \text{5, }\left[1\times \text{1, 256}\right]\times 2$$
CoordAtt (1)	$56\times 56$	$1\times \text{1, 8}$ $\left[1\times \text{1, 256}\right]\times 2$
Layer2	$28\times 28$	$$\left[\begin{array}{c}1\times \text{1, 9}\\ 1\times \text{1, 4}\end{array}\right]\times \text{5, }\left[1\times \text{1, 512}\right]\times 2$$
CoordAtt (2)	$28\times 28$	$1\times \text{1, 16, }\left[1\times \text{1, 512}\right]\times 2$
Layer3	$14\times 14$	$$\left[\begin{array}{c}1\times \text{1, 17}\\ 1\times \text{1, 4}\end{array}\right]\times \text{5, }\left[1\times \text{1, 1024}\right]\times 2$$
CoordAtt (3)	$14\times 14$	$1\times \text{1, 32, }\left[1\times \text{1, 1024}\right]\times 2$
Layer4	$7\times 7$	$$\left[\begin{array}{c}1\times \text{1, 33}\\ 1\times \text{1, 4}\end{array}\right]\times \text{5, }\left[1\times \text{1, 2048}\right]\times 2$$
Maxpooling	$1\times 1$	$7\times 7\text{, stride 7}$
Params		$4.54\times 1{0}^{6}$
FLOPs		$538.93\times 1{0}^{6}$

The full-dimensional dynamic convolution residual module extracts features from CXRs and enhances feature extraction by learning the attention weights of the convolution kernels in four dimensions: input channel, output channel, convolution kernel space, and number of convolution kernels, which improves the classification accuracy of the network while reducing the number of parameters. The FCA module decomposes the channel attention into two one-dimensional feature-encoding processes using CA. It extracts relevant feature information from both horizontal and vertical directions and aggregates them. This allows the network to obtain interchannel information while preserving the position information related to the direction, thereby improving the ability of the network to extract the features of lesion areas. Again, the obtained feature information is input into the global-average pooling layer to unify the image size. Finally, the extracted features are classified using Softmax.

### ODConv ResBolck

A regular convolution layer has a single static convolution kernel applied to all the input samples. A dynamic convolution layer uses a linear combination of $n$ convolution kernels dynamically weighted by an attention mechanism, which makes the convolution operation dependent on the input image. However, existing studies assign dynamic properties to the convolution kernel only through the number of convolution kernels, ignoring information about the three dimensions of the convolution kernel: the spatial size of the convolution kernel, number of input channels, and number of output channels. In particular, the dynamic convolution layer can continuously and automatically adjust the convolution parameters in the spatial and channel dimensions. Compared to static-convolutional methods, the dynamic adaptation of the dynamic convolution layer offers the following advantage in feature extraction: enhanced feature-extraction capability, as it is capable of adjusting the size and number of convolution kernels in accordance with the dimensions of the input feature maps and number of channels. Consequently, for a given input image, the weights of each convolution kernel share the same attention scalar, which limits their ability to capture rich contextual cues. Subsequently, in the convolution layer, replacing a regular convolution with a dynamic convolution increases the number of convolution parameters by a factor of $n$. When dynamic convolution is performed on many convolution layers, the number of model parameters significantly increases.

**Fig. 6:**
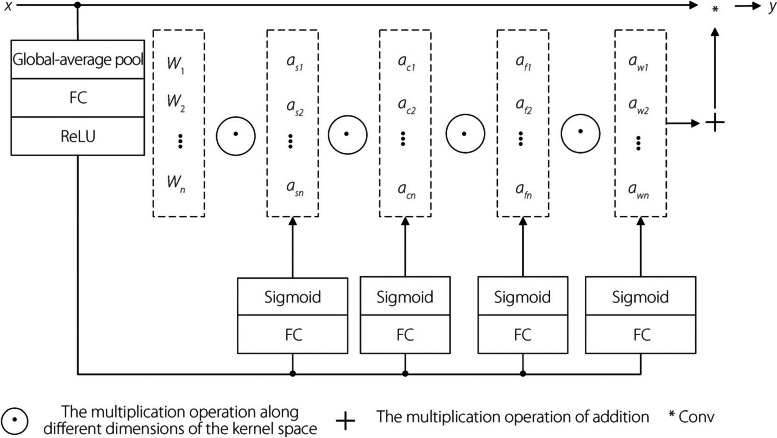
ODConv structure

Subsequently, ODConv [24] solves these problems. The ODConv module uses a novel multidimensional attention mechanism with a parallel strategy that learns the four types of attention in a convolution kernel in a parallel manner, along the four dimensions of the convolution kernel space. The four types of attention learned by ODConv are complementary. The corresponding convolution kernels gradually incorporate them, significantly enhancing the feature-e*x*traction ability of the basic convolution operation of the CNN while also reducing the number of network parameters. The structure of ODConv is shown in Fig. 6.

First, the input $x$ is compressed into a feature vector with ${c}_{in}$ length by a channel-by-channel global-average pooling-layer operation. The FC layer maps the compressed feature vector to a low-dimensional space with a reduction ratio of $\gamma$. Each of the four head branches on the right side of the figure contains a FC layer with output sizes $k\times k$,${c}_{in}\times 1$, and $n\times 1$, and a sigmoid activation function that performs a normalization operation on the compressed feature vectors to generate ${\alpha }_{si}$, ${\alpha }_{ci}$,${\alpha }_{fi}$, and ${\alpha }_{wi}$, respectively, and shares ${\alpha }_{si}$, ${\alpha }_{ci}$, ${\alpha }_{fi}$, and ${\alpha }_{wi}$ to all convolution kernels, respectively. This, in turn, enhances the feature-extraction capability of the network for the input chest slices.

**Fig. 7:**
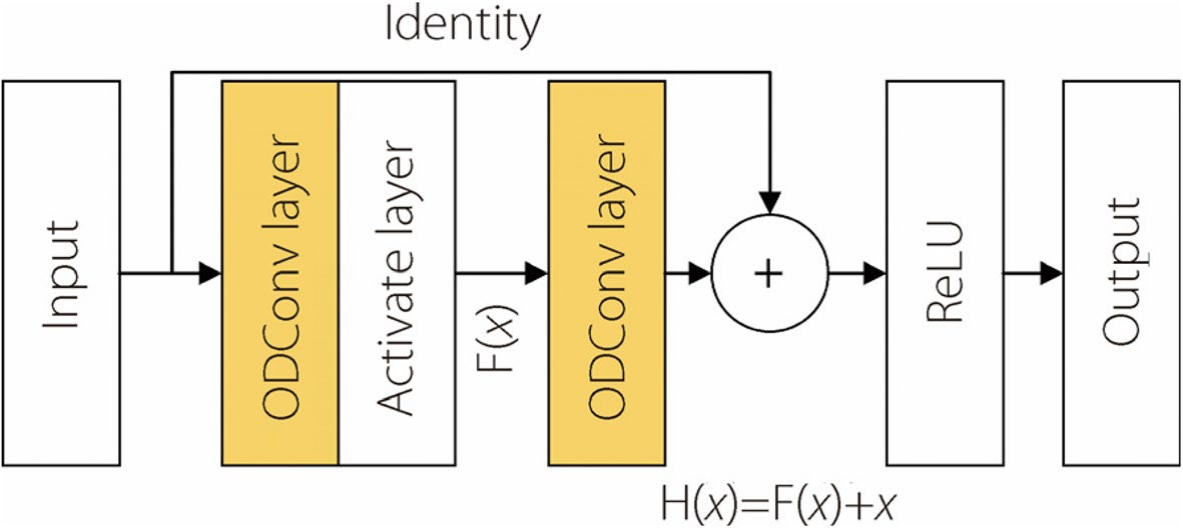
ODConv ResBolck module structure

Therefore, in this subsection, ODConv is used to replace static convolution in the residual module in the network. The structure of the improved ODConv ResBolck module is shown in Fig. 7, where the yellow part is the ODConv residual module.

### FCA module

**Fig. 8:**
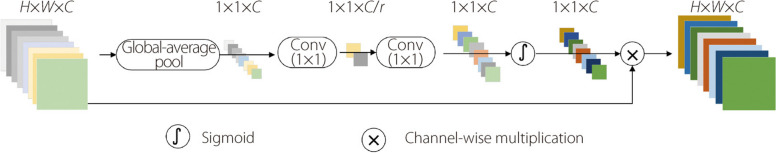
CA module structure

The attention mechanism is widely used in neural network models, and many networks have adopted the SE attention-mechanism module. However, the module only extracts information related to the channel, ignoring the effect of location information on the output. The CBAM module introduces spatial information encoding through a large-scale convolution, considering both the channel and spatial dimensions. It then combines the extracted information to obtain attention feature maps containing both channel and spatial information. However, the convolution in the CBAM module can only extract local relations and cannot extract long-distance relations. Therefore, in this study, the CA module is adopted [25], which can encode the horizontal and vertical position information into the channel attention so that the mobile network can pay attention to a large range of position information without requiring excessive computation. The structure of the CA module is illustrated in Fig. 8.

In the CA module, the global pooling operation is decomposed into two one-dimensional feature vectors for the encoding operation, and the input CXR with dimensions *C*×*H*×*W* is pooled into X and Y to generate feature maps with dimensions *C*×*H*×1 and *C*×1×*H*, respectively. The CA module comprises compression and excitation segments. The compression operation is performed on the input image using the global pooling operation, where the input feature images $x\in {R}^{H\times {\mathbb{W}}\times C}$ are averaged by the channel to generate $z\in {R}^{H\times {\mathbb{W}}\times C}$, etc. For example, in channel *C*,

1 $${z}^{c}={F}_{gp}\left({x}^{c}\right)=\frac{1}{H\times W}{\sum }_{i=1}^{H}{\sum }_{j=1}^{W}{x}^{c}(i,j),1\le c\le C$$

In Eq. (1), ${F}_{gp}(\cdot)$ denotes the global pooling operation, $H$ denotes the height of the input feature $x$,$W$ denotes the width of the input feature $x$, and $C$ denotes the number of channels of the input feature $x$. The channel weight $s$ is then calculated as follows:

2 $$s=f\left({F}_{sq2}\left(\delta \left({F}_{sq1}((z))\right)\right)\right)$$

In Eq. (2), $f$ denotes the Sigmoid activation function, $\delta$ denotes the ReLU linear correction unit, and ${F}_{sq1}(\cdot)$ and ${F}_{sq2}(\cdot)$ represent the 1 × 1 convolution layer with channel-reduction parameter $r$. Finally, the inputs and obtained weight $s$ are multiplied according to the channel, where $\left[{s}^{1},{s}^{2},\cdots ,{s}^{C}\right]$, to obtain

3 $${\widetilde{x}}^{c}={s}^{c}{x}^{c},1\le c\le C$$

In Eq. (3), the output features $\widetilde{x}=\left[{\widetilde{x}}^{1},{\widetilde{x}}^{2},\cdots ,{\widetilde{x}}^{C}\right]$ of the CA have the same dimensions as the input features $x\in {R}^{H\times {\mathbb{W}}\times C}$.

**Fig. 9:**
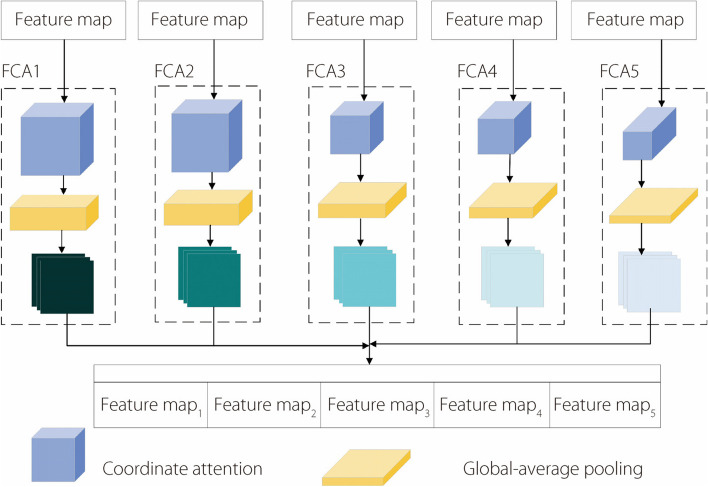
FCA module structure

Additionally, the FCA module based on the CA can aggregate deep and shallow features in the network, which mainly comprise the CA module and global-average pooling layer. First, this module performs fusion processing of the features extracted by the CA module. The image size is then unified to 1×1 using a global-average pooling operation. Finally, the obtained feature-fusion results are input into a deep network for feature aggregation. The structure of the FCA module is illustrated in Fig. 9.

### Loss function

The loss function estimates the model by measuring the difference between the predictions of the model $f(x)$ and ground truth $y$. This subsection describes the use of a cross-entropy loss function in the classification method. To ensure that the input to cross-entropy is a probability distribution, a Softmax function is added before the cross-loss function. This process transforms the network output into a probability distribution within the range [0, 1]. The equation is as follows:

4 $$Loss=-{\sum }_{i=1}^{C}{y}_{i}{\mathit{log}P}_{i}$$

where ${P}_{i}$ denotes the output probability and ${y}_{i}$ denotes the true category. Equation (4) shows that the value of $Loss$ is always positive and $Loss$ tends to 0 as ${P}_{i}$ tends to ${y}_{i}$.

## Results

### Datasets

Due to the specificity of medical datasets, problems such as sample imbalance, low data quality, and dataset scarcity can occur. The datasets used in this study were derived from two publicly available pneumonia datasets. The first was the COVID-19 Radiography Database developed by Chowdhury et al. [26]. It contains images of COVID-19 positive, normal, and common pneumonia [26, 27]. The second was CXR (Covid-19 & Pneumonia), a Kaggle database collected from various publicly available resources [28–30] containing three categories of chest radiographs: common pneumonia, COVID-19, and normal.

By combining the above two datasets, the dataset used in this study contained 12,880 chest radiographs from three categories of images: COVID-19, normal pneumonia, and normal. The same preprocessing operations were performed on the dataset to ensure uniformity of the data formats. The division ratio of the experimental datasets used in this study was 7:2:1.

To ensure a fair comparison, the same training strategy is adopted and the network is implemented using Pytorch1.10.1. The CPU processor used in this experiment was an Intel I9-11900K processor with 64GB of RAM, and the GPU was an Nvidia RTX A4000 (16G).

### Experimental results and analysis

To verify the effectiveness of the proposed method, eight networks, DarkCovidNet [31], ConvNext [32], ShuffleNet [33], MobileNetv2 [34], ResNet18, ResNet50, ResNet101, and EfficientNet [35], are selected as the comparison networks. By comparing the classification effects of different networks, the effectiveness of the improved method is demonstrated. In this subsection, the experimental results are analyzed in two parts: training and testing sets.

1. Analysis of validation results of the training set

**Fig. 10:**
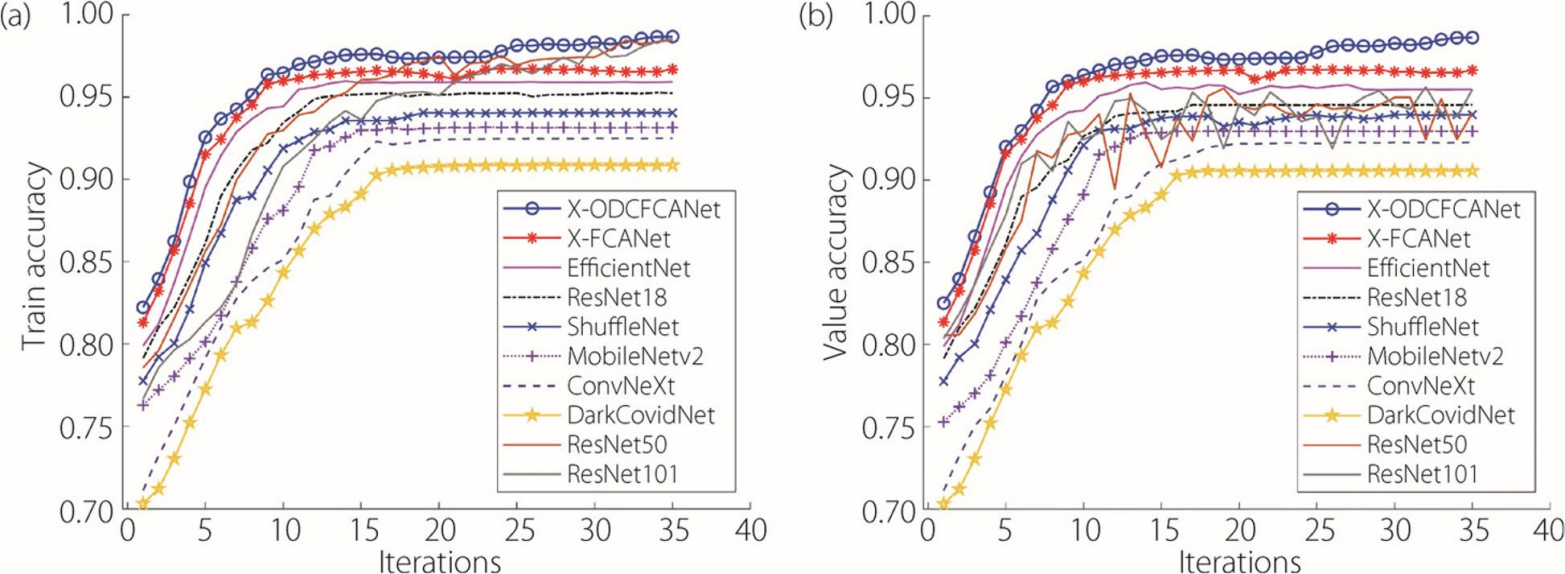
Accuracy-variation curves for each classification method on the training (**a**) and validation (**b**) sets

After 35 iterations with a fixed learning rate and cross-quadratic fold validation, the accuracy-change curves of each classification method for the training and validation sets are obtained, as shown in Fig. 10. The figure shows that the accuracy of each model gradually tends to be smoothed with the number of iterations. The accuracy rate of this method is higher than that of the other eight networks in both the training and validation sets. The experimental results show that the addition of the FCA and ODConv modules can improve the classification accuracy of the model.

In this subsection, X-ODFCANet is trained, and after obtaining the experimental results, the average accuracy and number of parameters after four-fold cross-validation on the training set are compared with those of the eight models, as shown in Table 2. From Table 2, after adding the FCA and ODConv modules, the accuracy of the model is improved by 3.77%, and the number of parameters is decreased to 4.45M, which proves the effectiveness of the improved method in this study.

**Table 2:** Comparison of the average accuracy of each model in the training set

Model	Accuracy	FLOP	Parameter
DarkCovidNet	90.23%	2123.26M	14.53M
ConvNeXt	92.16%	4454.77M	27.80M
MobileNetv2	92.86%	**151.69M**	**1.26M**
ShuffleNet	93.40%	362.27M	2.23M
ResNet18	94.56%	1823.52M	11.20M
EfficientNet	95.03%	401.92M	4.01M
ResNet50	94.97%	4137.70M	23.51M
ResNet101	93.62%	7864.39M	42.51M
Proposed	**96.27**%	538.93M	4.45M

(2) Analysis of experimental results for testing set after the training was completed. The optimal weights of the improved method were obtained. The model was predicted on the testing set to obtain the precision, recall, and specificity indices of X-ODFCANet for the prediction of each category, and the testing results were compared with those of the eight models. The final metrics of the experimental results of the nine models for the three categories of chest radiograph classification, namely, COVID-19, common pneumonia, and normal, are shown in Tables 3, 4, and 5, respectively. These three tables show that the proposed method outperforms the other methods in all three categories, except for the general category. This proves that the FCA and ODConv modules can improve classification accuracy and reduce the number of parameters during training.

**Table 3:** Classification results of COVID-19 for each model

Model	Precision	Recall	Specificity
DarkCovidNet	0.896	0.903	0.912
ConvNeXt	0.922	0.914	0.992
ShuffleNet	0.940	0.940	0.991
MobileNetv2	0.979	0.914	0.995
ResNet18	0.981	0.923	0.996
EfficientNet	**0.991**	0.966	**0.999**
ResNet50	0.974	0.966	0.997
ResNet101	**0.991**	0.948	**0.999**
Proposed	**0.991**	**0.991**	**0.999**

**Table 4:** Classification results of common pneumonia for each model

Model	Precision	Recall	Specificity
DarkCovidNet	0.903	0.912	0.909
ConvNeXt	0.935	0.956	0.868
ShuffleNet	0.936	0.971	0.868
MobileNetv2	0.974	0.929	0.952
ResNet18	**0.982**	0.946	0.965
EfficientNet	0.946	0.982	0.889
ResNet50	0.972	0.940	0.947
ResNet101	0.961	0.967	0.921
Proposed	0.980	**0.984**	**0.961**

**Table 5:** Classification results of each model for the normal category

Model	Precision	Recall	Specificity
DarkCovidNet	0.906	0.913	0.922
ConvNeXt	0.883	0.833	0.964
ShuffleNet	0.926	0.883	0.978
MobileNetv2	0.811	0.934	0.929
ResNet18	0.851	0.956	0.945
EfficientNet	0.948	0.858	0.985
ResNet50	0.855	0.934	0.949
ResNet101	0.908	0.905	0.970
Proposed	0.956	0.947	0.986

**Fig. 11:**
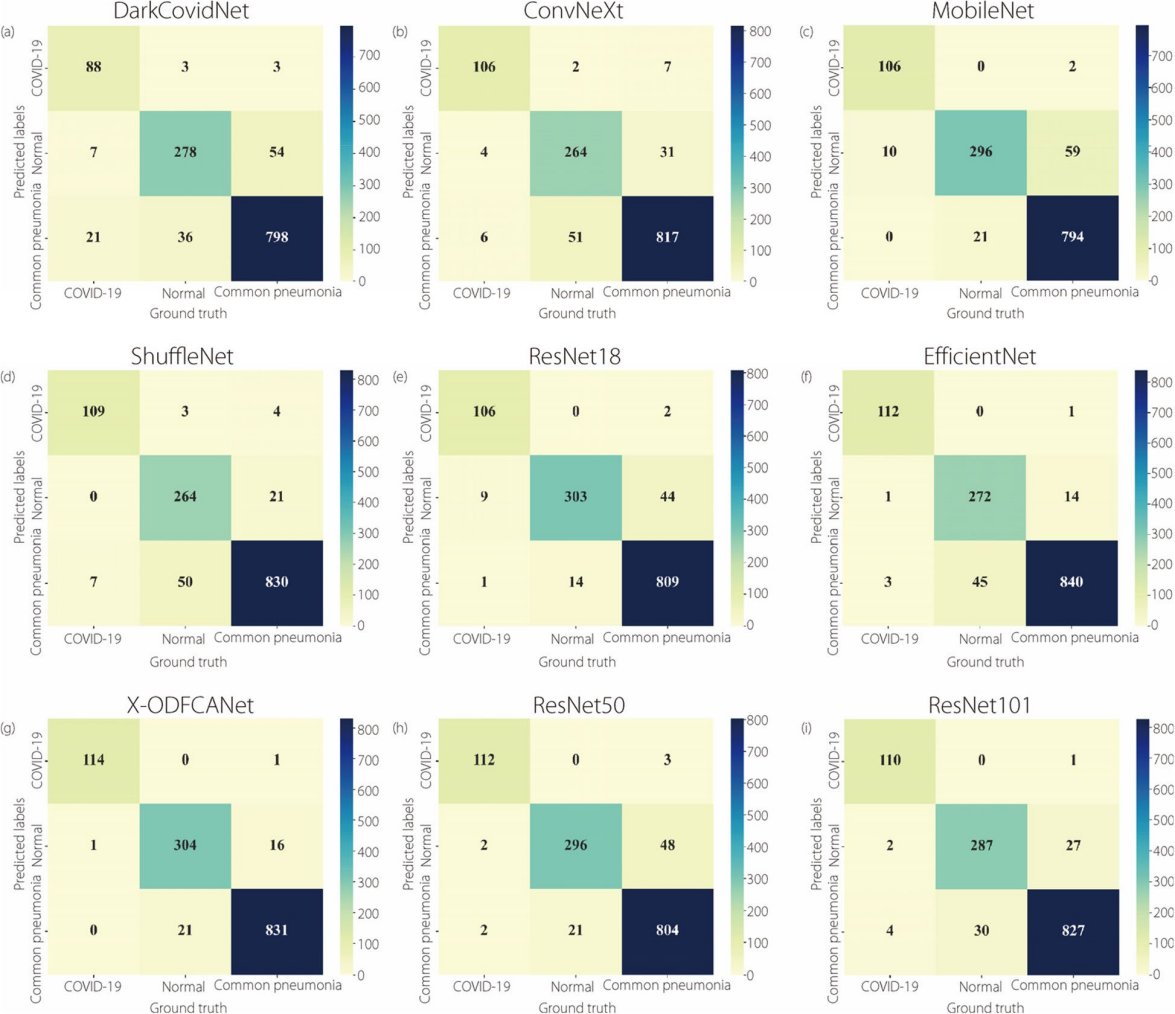
Confusion-matrix diagram for nine models

To compare the classification of the improved method proposed in this study for pneumonia more intuitively, the confusion matrices of the nine models are plotted to compare the effects of the FCA module and ODConv on the model-performance improvement. The observation of Fig. 11g shows that the average accuracy of X-ODFCANet for the prediction of COVID-19, normal, and pneumonia is 97.57%, which is an increase of 3.77%, proving the effectiveness of the proposed method.

## Discussion

### Qualitative analysis

The experimental results, presented in Tables 3, 4, and 5, indicate that the network exhibits misclassification errors on a subset of chest radiographs displaying inconspicuous symptoms. Furthermore, the classification accuracy is not balanced across different categories. This may be because the chest radiographs exhibiting inconspicuous symptoms show similarities to chest radiographs belonging to other categories in terms of image features; thus, classifying the data accurately using the network is challenging.

Furthermore, the issue of network inconsistencies across categories should be addressed in future studies. In practice, the number of samples in different categories may vary considerably, which may result in the suboptimal performance of the model in a few categories. Therefore, new loss functions and training strategies must be designed to address these issues. Although the method proposed in this study has better accuracy and fewer model parameters, it is more computationally intensive. In future studies, the feature-extraction method will be further improved to solve this problem.

As illustrated in Fig. 11, the efficacy of our method in classifying the data was demonstrated to be excellent across all three categories. However, this method encounters difficulties in distinguishing between normal and ordinary lungs. This is because of the lack of a clear distinction between the features of ordinary and normal lungs. In the future, the construction of multitasking learning strategies or the utilization of more effective data preprocessing and enhancement techniques will help resolve this shortcoming. The number of samples may vary significantly among categories, resulting in a poorly performing model for a few categories. Therefore, novel loss functions and training strategies must be developed to address these issues. In future work, further optimization of the network structure will be beneficial for improving the performance and reducing the number of network parameters.

### Limitations

In practice, the number of samples may vary considerably among different categories; thus, the model may perform inadequately in a few categories. Therefore, novel loss functions and training strategies must be developed to address these issues. In the future, the feature-extraction method will be further improved to solve the problem of high model computation. Owing to the difficulty in manually labelling the current chest-film samples and the small amount of labelled data, weakly supervised or unsupervised learning can be used in the future to train the chest-film dataset containing only image labels.

The utilization of unsupervised learning methodologies such as self-supervised learning or generative adversarial networks [36] may be considered to train models utilizing unlabeled data. These methods may assist in optimizing the utilization of unlabeled data, thereby enhancing model performance. In addition, the potential of transfer learning or meta-learning may be investigated to enhance the generalization capacity of the model. These methods facilitate the use of existing knowledge across diverse tasks and domains, thereby enhancing the efficacy of the model. Although the proposed method demonstrates satisfactory performance in certain respects, numerous challenges require further attention. In future endeavors, the model performance will be further enhanced.

The X-ODFCANet method can be applied to other medical-imaging tasks such as the classification of other types of image data and other chest diseases. Because X-ODFCANet has been demonstrated to be well adapted to lung structures, it can be laterally generalized following parameter tuning for similar lung diseases, such as lung cancer. These applications serve to validate the generality and scalability of the proposed method. Furthermore, the feature-extraction method of X-ODFCANet can be enhanced by designing new feature-extraction networks to enhance its feature-extraction capability. In the future, the possibility of developing new model structures or adopting more efficient model structures will be investigated, such as lightweight or deeply separable structures. These enhancements are anticipated to further optimize the performance of our method for medical-image-processing tasks.

However, this study did not provide a detailed classification of pneumonia, which is necessary for precise treatment protocols. The proposed method did not differentiate between bacterial and viral pneumonia, even though different types of pneumonia require different treatments. In future studies, a more fine-grained classification of pneumonia should be considered to enable more accurate treatment protocols.

## Conclusions

To address the issues of low accuracy and a high number of model parameters in pneumonia-classification recognition, X-ODFCANet, an ODConv feature-coordinated attention-classification network for CXRs was proposed. ODConv replaces the original static convolution module and applies four dimensions to the input CXRs for feature extraction: the spatial dimensions of the convolution kernel, number of input channels, number of output channels, and number of convolution kernels. The feature information extracted from the four dimensions was fused, and the extracted features were classified. Compared with static convolution, ODConv improves feature-extraction accuracy and reduces information redundancy, resulting in a more lightweight model with fewer parameters. In addition, the FCA module fuses the feature information obtained from the horizontal and vertical spatial-direction aggregations. The image size is then unified using a global-average pooling operation. The feature-fusion results are ultimately inputted into a deep-learning network for classification. The FCA module improves the ability of the model to extract feature information, resulting in more accurate pneumonia classification.

Experiments were conducted on the dataset used in this study using an improved classification network. The results showed that X-ODFCANet effectively improved the accuracy of the pneumonia-classification method and reduced the number of model parameters. The average accuracy of the three chest radiographs also increased. The average accuracy of the pneumonia-classification method for COVID-19, common pneumonia, and normal cases was 97.57%. This was 3.77% higher than the accuracy of ResNet18. These results demonstrate that X-ODFCANet can effectively enhance the accuracy of pneumonia classification while reducing the number of model parameters.

## Data Availability

The public datasets used in this study are publicly available COVID-19 Radiography Database provided online as well as Chest X-ray (Covid-19 & Pneumonia).

## Abbreviations

COVID-19: Corona virus disease 2019
CXR: Chest X-ray
CT: Computed tomography
MRI: Magnetic resonance imaging
SE: Squeeze-and-excitation
FC: Fully connected
FCA: Feature coordinate attention
ODConv: Omni-dimensional dynamic convolution
CA: Coordinate attention
CBAM: Convolution block attention module
ReLU: Rectified linear unit
