# Correction: Omni-dimensional dynamic convolution feature coordinate attention network for pneumonia classification

**DOI:** 10.1186/s42492-024-00170-x

**Published:** 2024-07-23

**Authors:** Yufei Li, Yufei Xin, Xinni Li, Yinrui Zhang, Cheng Liu, Zhengwen Cao, Shaoyi Du, Lin Wang

**Affiliations:** 1 School of Information Science and Technology, Northwest University, Xi’an, 710127 Shaanxi Province China; 2 Department of Ultrasound, the Second Affiliated Hospital of Xi’an Jiaotong University, Xi’an, Shaanxi Province 710004 China; 3 National Key Laboratory of Human-Machine Hybrid Augmented Intelligence, National Engineering Research Center for Visual Information and Applications, and Institute of Artificial Intelligence and Robotics, Xi’an Jiaotong University, Xi’an, Shaanxi Province 710004 China

**Correction:** **Vis. Comput. Ind. Biomed. Art 7, 17 (2024)**

**https://doi.org/10.1186/s42492-024-00168-5**

Following publication of the original article [1], the authors reported a typesetting error in the authors’ affiliations.

Due to a typesetting error, the affiliations of authors Shaoyi Du and Lin Wang were mistakenly assigned.

The originally published version:

Shaoyi Du^1,2^ and Lin Wang^3^

The correct version should read:

Shaoyi Du^2,3^ and Lin Wang^1^

The original article [1] has been updated.
